# Textaphrenia: psychological repercussions among young adults

**DOI:** 10.1192/j.eurpsy.2023.2119

**Published:** 2023-07-19

**Authors:** A. H. I. Abu Shehab, I. Fulga, V. Gheorman, S. Trifu, A. B. Ciubară, S. L. Burlea, A. Ciubară

**Affiliations:** 1 Psychiatry, “Elisabeta Doamna” psychiatry hospital; 2 Forensic medicine, „Dunărea de Jos” University of medicine, Galati; 3 Psychiatry, University of Medicine and Pharmacy of Craiova, Craiova; 4 Psychiatry, Carol Davila University of Medicine and Pharmacy, Bucharest; 5 Orthopaedics and Traumatology, „Dunărea de Jos” University of medicine, Galati; 6 Dental Medicine, University of Medicine and Pharmacy “Grigore T. Popa”, Iasi; 7 Psychiatry, „Dunărea de Jos” University of medicine, Galati, Romania

## Abstract

**Introduction:**

In the past ten years, the use of mobile phones for text messaging has increased dramatically. This is due to its utility in communication and interactions in the professional and personal spheres. Recent studies have demonstrated that the usage of text messaging via smart phones for social networking has also contributed to the reduction of loneliness. However, excessive usage of mobile phones is linked to certain psychiatric morbidities. Textaphrenia is one of these newly emerging mental health issues.

**Objectives:**

The aim of this review is to analyze the effects of excessive use of mobile phones among young population.

**Methods:**

Medical publications, studies, and specialized information on the subject were used to complete this work.

**Results:**

Researchers have shown a correlation between excessive cell phone use and personality traits like neuroticism, extraversion, low self-esteem,and impulsivity. In addition to sleep disturbances, anxiety, tension, and depressed mood are all symptoms that have been linked to unhealthy cell phone use. These symptoms have also been linked to abusive use of the internet. Moreover, the current investigation sheds light on the coexisting link that exists between problematic cell phone use and the use of substances like tobacco and alcohol.

**Conclusions:**

The evidence suggests that excessive usage of smart phones for text messaging might lead to the development of psychological dependence and can interfere with daily tasks.

**Disclosure of Interest:**

None Declared

